# Research Progress of Titanium-Based High Entropy Alloy: Methods, Properties, and Applications

**DOI:** 10.3389/fbioe.2020.603522

**Published:** 2020-11-11

**Authors:** Ning Ma, Shifeng Liu, Wei Liu, Lechun Xie, Daixiu Wei, Liqiang Wang, Lanjie Li, Beibei Zhao, Yan Wang

**Affiliations:** ^1^School of Metallurgical Engineering, Xi’an University of Architecture and Technology, Xi’an, China; ^2^Hubei Key Laboratory of Advanced Technology for Automotive Components, Wuhan University of Technology, Wuhan, China; ^3^Institute for Materials Research, Tohoku University, Sendai, Japan; ^4^State Key Laboratory of Metal Matrix Composites, School of Material Science and Engineering, Shanghai Jiao Tong University, Shanghai, China; ^5^Chengsteel Group Co., Ltd., HBIS Group Co., Ltd., Chengde, China

**Keywords:** titanium-based high entropy alloy, biomedical application, implant, complex alloys, multi-principal element alloys

## Abstract

With the continuous progress and development in the biomedicine field, metallic biomedical materials have attracted the considerable attention of researchers, but the related procedures need to be further developed. Since the traditional metal implant materials are not highly compatible with the human body, the modern materials with excellent mechanical properties and proper biocompatibility should be developed urgently in order to solve any adverse reactions caused by the long-term implantations. The advent of the high-entropy alloy (HEA) as an innovative and advanced idea emerged to develop the medical implant materials through the specific HEA designs. The properties of these HEA materials can be predicted and regulated. In this paper, the progression and application of titanium-based HEAs, as well as their preparation and biological evaluation methods, are comprehensively reviewed. Additionally, the prospects for the development and use of these alloys in implant applications are put forward.

## Introduction

In recent decades, bio-medical materials are widely used in implants and repair surgeries due to their high strength, wear, corrosion resistance, and biocompatibility (Saini, 2015). In order to improve the bone tissue rehabilitation in biomedical applications, a kind of materials should be selected that possess similar properties to the natural bone so it can maintain the cell adhesion function after implantation, promote the tissue repair, and accelerate the healing process. Among all the biomedical implant materials, metallic ones are the most widely used material group in the clinical practices. The metallic biomaterials include stainless steel, CoCrMo alloy, NiTi shape memory alloy, magnesium alloy, titanium, and its alloys (Saini, 2015; Liu et al., 2020; Wang W. et al., 2020). Titanium and its traditional alloys are ideal biomedical materials with good mechanical properties, biocompatibility, and corrosion resistance. These materials are mostly used in orthopedics and dental implants (Niinomi, 2003, 2008; Geetha et al., 2009; Zhu et al., 2016; Zhang et al., 2017; Rabadia et al., 2018), such as plates, stents, hip and knee joint replacements, dental roots, etc. However, pure titanium and its alloys also have some limitations such as poor wear resistance in which the material wears out and produces some metallic particles and debris under long-term and repeated stress conditions. These metallic particles and debris can cause local tissue lesions and inflammation. The Ti-6Al-4V and Ti-6Al-7Nb alloys are known as the traditional Ti alloys (Kobayashi et al., 1998; Tamilselvi et al., 2006; Assis and Costa, 2007; Ding et al., 2016; Wang H. et al., 2019). These two materials fulfill the strength requirement of the implant, but the alloying elements such as the Al and V can cause toxic effects and adversely influence the live tissues and organs. The Al element can accumulate in the brain, liver, spleen, kidney, thyroid, and other tissues and organs and cause some degree of damages (Attarilar et al., 2020). Also, the V element may cause bone softening, anemia, and nerve disorders. Moreover, the elastic modulus of these two titanium alloys (about 120 GPa) is still very high compared to natural bone (10∼35 GPa). Niinomi (2008) found that titanium alloys with low elastic modulus have better load transfer characteristics than the alloys with high elastic modulus. The dense metal implant bears most of the applied load and leads to a significant reduction of stress level in the periphery of bone tissue, hence the density and strength of the bone tissue gradually decrease. This phenomenon is known as the stress shielding effect, which causes slow bone healing, bone resorption, implant loosening, and failure.

Recently, high-entropy alloys (HEAs) are known as novel metallic functional materials and they attract considerable attention. HEAs have great potential in the field of biomedicine and seem to finds lots of applications in the medical industry (Yan and Zhang, 2020). The concept of HEA was originated from increasing the number of elements to increase the mixing entropy of the material to achieve the purpose of a stable solid solution alloy formation. They are also called multi-principal element alloys (MPEAs) or compositionally complex alloys (CCAs) (Miracle and Senkov, 2017; George et al., 2020). Based on the multiple element principal, HEAs are the representative of a new class of alloys that provide better performance through composition adjustment and the controlling methods, in which the phase composition encountered some transitions from a single-phase solid solution toward a variety of complex phase compositions. Moreover, researchers have divided a large number of HEAs into two main categories and analyzed their deformation mechanisms (Brechtl et al., 2020). The first one is based on the crystallographic structure of the phase and includes FCC-based, BCC-based, HCP-based, amorphous, and intermetallic HEAs. The second one is categorized according to the phase types and includes single-phase, dual-phase, eutectic and multi-phase HEAs. The HEAs had attracted substantial attention due to their excellent properties, such as high strength/hardness, high wear resistance, high fracture toughness, excellent low temperature performance and structural stability, good corrosion resistance, oxidation resistance, etc. (Juan et al., 2015; Xu et al., 2015; Shang et al., 2017; Chen J. et al., 2018; Jin et al., 2018; Qiu, 2018; Sharma et al., 2018; Shuang et al., 2019; Tian et al., 2019; George et al., 2020; Yao et al., 2020). For instance, Prùša et al. (2020) investigated the strengthening mechanism of ultrafine-grained CoCrFeNiNb HEA by mechanical alloying and spark plasma sintering that showed ultra-high strength of 2412 MPa and high hardness of 798 ± 9 HV at 1000°C. Furthermore, researchers have also realized that rapid oxidation under high temperature conditions limits the high-temperature applicability of HEAs. The addition and content of alloying elements are the key factors affecting the oxidation resistance and application of HEAs. Recent research have confirmed that the addition of Al and Cr to the alloy can effectively improve the oxidation resistance of HEAs, and the formation of some complex oxides can also provide better protection for the alloy (Waseem and Ryu, 2020).

This new type of alloy and its modern concept breaks the bottleneck of traditional material design and introduce new ideas for the research and development of high-performance metallic materials. Therefore, the design of Ti-HEAs materials with excellent biocompatibility and good mechanical properties is of great significance for the advancement of medical implants.

## Emergence and Development of Titanium-Based HEAs

Yeh et al. (2004) and Yeh (2006) introduced the HEAs in 2004, this new class of metallic materials usually composed of 5 or more metallic elements in nearly equiatomic proportions (Cantor et al., 2004; Miracle and Senkov, 2017; Zhang et al., 2018). Although the composition of high entropy alloys is very complex, it is usually composed of a single phase or two phase structures with proper stability and flexibility (George et al., 2020).

The HEA design is based on the four effects, including the high-entropy effect, the lattice distortion, sluggish diffusion, and the “cocktail” effect (Zhang et al., 2014). The high entropy effect is the landmark concept of HEAs. Initially, scholars believe that the alloy which contains the multi-principal elements will be led to the production of various intermetallic compounds and or complex microstructures. However, after a while, they have discovered a special phenomenon in which HEAs do not form a large number of intermetallic compounds, rather they tend to form a simple BCC, HCP, and FCC phase or even amorphous structures after solidification, this phenomenon is defined as the high-entropy effect. Due to this high-entropy effect, the high mixing entropy strengthens the mutual dissolution between elements and thereby hindering the formation of intermetallic compounds. In general, HEAs include five or more elements that each of them has the same probability of occupying lattice points. The difference in atomic sizes during the formation of the solid solution will cause lattice distortion in the crystal structure, which is called the lattice distortion effect. When the lattice distortion energy is too high, the crystal cannot maintain a stable structure, and the distorted lattice will collapse to form an amorphous phase or intermetallic compounds. But, whether in crystal or amorphous state, this distortion effect will affect the mechanical properties, electrical properties, optical properties, and even chemical properties of the material. The sluggish diffusion effect is attributed to the fact that in the casting process of HEAs, the coordinated diffusion of multiple elements gets more difficult due to the liquid-solid phase changes, and severe lattice distortion will slow down the diffusion rate of the elements. Therefore, the phase separation rate at high temperature is slow, even suppressed at low temperatures, which is the major cause of nano-precipitate formation in the as-cast HEAs. The “cocktail” effect refers to a combination effect due to the multi-element interaction of the HEAs, which combines the original characteristics of various elements and relatively eliminates their shortcomings. Considering the “four core” effect, it is easy to obtain solid solution phases, nanostructures, and even amorphous structures with high thermal stability in HEAs (Yeh, 2006; Zhang et al., 2014), and they can be regarded as the composite materials on the atomic scale. Moreover, the HEA design is not a simple mixing of elements, it is necessary to consider the interaction between elements that will affect the overall performance of the obtained alloy (George et al., 2020). At present, the HEA design firstly is done through computer simulations in which the influence of thermodynamics and kinetics of the alloy needed to determine, also the impact of phase formation laws must be considered (Koval et al., 2019). Computer simulation generally uses first-principle calculations and phase diagram calculations to predict the structure, phase stability, and mechanical properties of HEAs. Ge H. et al. (2017) used the first principle calculations to predict the elastic and thermal properties of ternary or quaternary refractory HEAs containing Al, Ti, V, Cr, Nb, and Mo elements. They found that the calculated properties by simulations are consistent with the experimental results. In recent years, the Hume-Rothery criterion and the valence electron concentration criterion are often used to study the phase and formation possibility of HEAs. Yao et al. (2017) used the three parameters Ω, δ, and ΔH of common solid solutions, intermetallic compounds, and NbTaV- (Ti, W) series alloys to predict the phase formation of the designed alloy.

**FIGURE 1:**
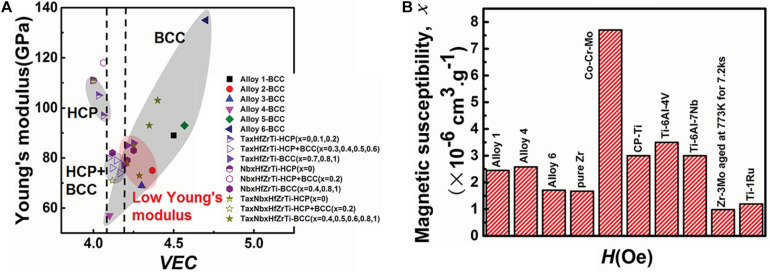
Performance comparison of titanium-based HEAs and other metallic alloys, **(A)** Dependence of Young’s modulus and crystalline phase structure on VEC in all the HEAs investigated. **(B)** Comparison of magnetic susceptibilities of alloys 1, 4, 6, and pure Zr with the alloys commonly used in medical devices. (Alloy 1: Ti_25_Zr_25_Nb_25_Ta_25_; Alloy 2: Ti_31.67_Zr_31.67_Nb_31.66_Ta_5_; Alloy 3:Ti_35_Zr_35_Nb_25_Ta_5_; Alloy 4: Ti_45_Zr_45_Nb_5_Ta_5_; Alloy 5:Ti_21.67_Zr_21.67_Nb_21.66_Ta_35_; Alloy 6: Ti_15_Zr_15_Nb_35_Ta_35_) (Yuan et al., 2019). Reproduced from Yuan et al. (2019) with permission.

In the recent research reports, the most HEAs used in medical implants consist of refractory elements with non-toxic and hypoallergenic nature (Stiehler et al., 2008; Yurchenko et al., 2020). Biomedical HEAs usually employ Ti and non-toxic and hypoallergenic elements of group IV and group V as main components with the addition of Cu and Co elements on the matrix. Discovery of the latest three TiTaHf-based HEAs that showed significant biocompatibility in immersion experiments has demonstrated the potential of these novel HEAs can be utilized as long-term implant materials (Gurel et al., 2020). Among them, the presence of Nb and Zr elements have made enormous contributions to the enhancement of material corrosion performance. Yuan et al. (2019) prepared a series of TiZrHfNbTa HEAs with low modulus, good biocompatibility, and low magnetic susceptibility. In addition, they systematically analyzed and summarized the performance of HEAs with the addition of any element. It is found that Young’s modulus in HEAs is relatively easier to control than the traditional implant metals, and the comparison between Ti-based HEAs and other metallic biomaterials are indicated in Figure 1. It provides more possibilities to utilize HEAs as biomedical implant materials in the future. Ching et al. (2020) reported a theoretical modeling technique to predict the properties of HEAs; they analyzed and predicted the performance of thirteen biocompatible HEAs. Their proposed technology is based on the quantum mechanical measurements, total bond order density (TBOD), and partial bond order density (PBOD). It can deeply analyze the electronic structure and interatomic bonding of HEAs and has an important guiding significance for the design and application of medical HEAs in the future.

## Fabrication Methods of Titanium Based HEAs

The HEA preparation methods mainly include ingot metallurgy, powder metallurgy, selective laser melting, laser cladding, magnetron sputtering, etc. Among them, ingot metallurgy, powder metallurgy, and selective laser melting are the most used techniques to prepare the bulk HEAs, while laser cladding and magnetron sputtering methods are commonly used in order to prepare HEA thin films or coatings. The advantages and limitations of various HEA preparation methods are listed in Table 1.

**TABLE 1:** The advantages and limitations of HEAs preparations.

Preparation methods	Advantages	Limitations	References
Arc Melting	➢ Simple process ➢ Wide range of applications	➢ Exist casting defects such as composition segregation, coarse structure, internal shrinkage cavity etc.	Zhang J. et al., 2020
Powder Metallurgy	➢ Near net shape forming ➢ Higher utilization of material ➢ Uniform composition ➢ Save metals and reduce costs	➢ Poor toughness ➢ Higher die cost	Ge W. et al., 2017; Wang et al., 2017
Magnetron sputtering	➢ Film thickness can be controlled by adjusting sputtering parameters. ➢ Low requirements on target composition ➢ High sputtering rate, and low base temperature can obtain a dense film surface	➢ Higher preparation cost. ➢ Complex equipment ➢ Lower target utilization	Yan et al., 2018; Zhang et al., 2018
Laser cladding	➢ High melting and cooling rate ➢ Small heat affected zone ➢ Metallurgical combination of coating and substrate	➢ The cladding layer is easy to crack ➢ Uneven distribution of composition	Xingwu et al., 2018; Yan et al., 2018; Liu J. et al., 2019

### Preparation of Bulk HEAs

#### Arc Melting

**FIGURE 2:**
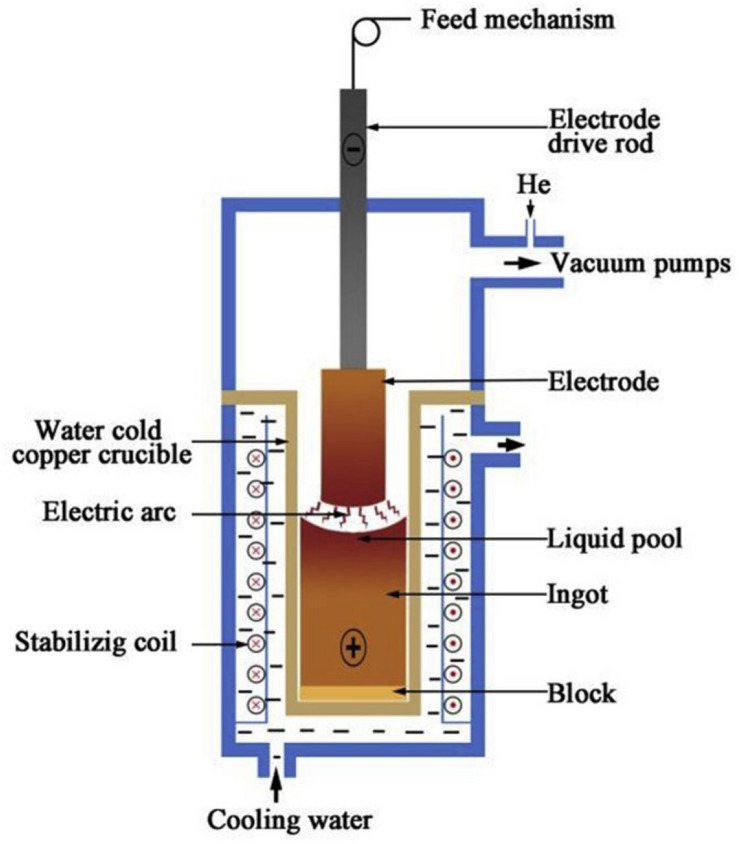
Schematic representation of the vacuum arc melting process (Feng et al., 2020). Reproduced from Feng et al. (2020) with permission.

Arc melting is currently one of the most commonly used preparation methods for the production of bulk HEAs (Baldenebro-Lopez et al., 2015; Chen Y. et al., 2018; Hou et al., 2019; Zhang J. et al., 2020). The process involves pouring a certain proportion of metallic materials into the tongs. Then, after repeatedly vacuuming, the vacuum furnace fills with the protective argon gas. Subsequently, the elements are completely melted by the plasma arc heating of the electrode, then the water-cooled rapid cooling process solidifies the whole melt into an alloy. The schematic of arc melting is shown in Figure 2.

**FIGURE 3:**
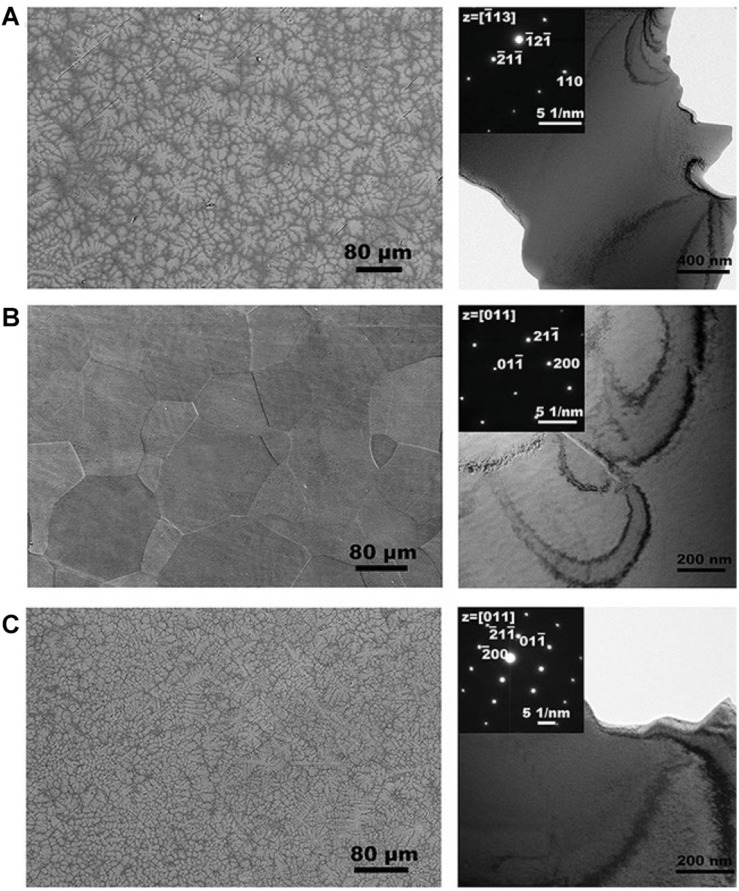
SEM images and TEM bright-field micrographs of three TiZrNbTa HEAs. **(A)** Ti_25_Zr_25_Nb_25_Ta_25_ **(B)** Ti_45_Zr_45_Nb_5_Ta_5_ **(C)** Ti_15_Zr_15_Nb_35_Ta_35_ HEA (Yuan et al., 2019). Reproduced from Yuan et al. (2019) with permission.

**FIGURE 4:**
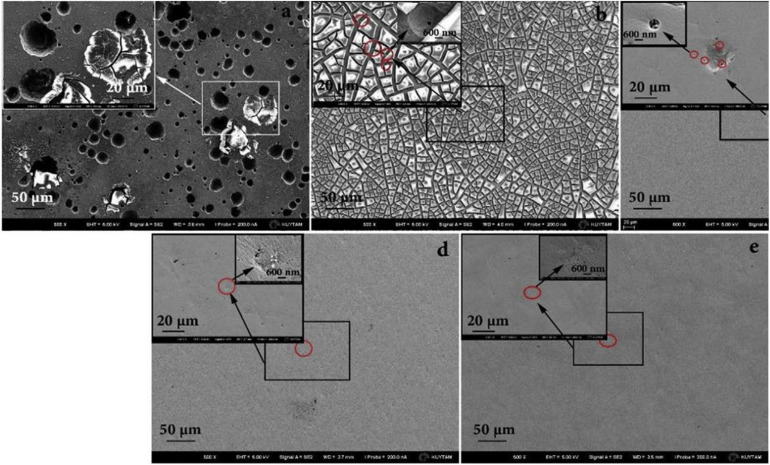
FESEM images of different alloys after the potentiodynamic tests in PBS electrolyte at 37°C. **(a)** 316L, **(b)** CoCrMo alloy, **(c)** Ti6Al4V, **(d)** TiZrTaHfNb HEA, and **(e)** Ti_1.5_ZrTa_0.5_Hf_0.5_Nb_0.5_ HEA (Motallebzadeh et al., 2019). Reproduced from Motallebzadeh et al. (2019) with permission.

Recently, Wang and Xu (2017) prepared a TiZrNbTaMo equiatomic HEA by arc-melting, which contains BCC1 and BCC2 dual phases, and it shows a good corrosion resistance in phosphate buffer solution. Dirras et al. (2016) explored the necking and fracture surfaces of the as-cast TiHfZrTaNb HEA, and they revealed a combination of multiple slip bands, grain boundary distortion, as well as shallow and deep dimples, which exhibited the high tensile plastic behavior. Yuan et al. (2019) developed the TiZrHfNbTa HEAs, and they focus on Ti_25_Zr_25_Nb_25_Ta_25_, Ti_45_Zr_45_Nb_5_Ta_5_, and Ti_15_Zr_15_Nb_35_Ta_35_ HEA. The transmission electron microscopy (TEM) and SEM images of three HEAs are shown in Figure 3. Typical dendritic morphology enriched with Nb and Ta are shown in Figure 3A,C, while the equiaxed grains is shown in Figure 3B. Furthermore, Ti_4__5_Zr_4__5_Nb_5_Ta_5_ HEA exhibited lower modulus values (57 Gpa) than the equiatomic TiZrHfNbTa HEA, which is similar to Young’s modulus of cortical bone (10∼30 Gpa). The mechanical properties of HEAs prepared by arc-melting are shown in Table 2. In addition, to further explore the corrosion resistance and wear resistance of the TiZrTaHfNb HEA, Motallebzadeh et al. (2019) made a comparison of TiZrTaHfNb and Ti_1.5_ZrTa_0.5_Hf_0.5_Nb_0.5_ HEA with 316L, CoCrMo, and Ti6Al4V alloys. The corrosion product morphologies of the samples after the electrochemical test in the phosphate buffer saline (PBS) electrolyte are shown in Figure 4. It can be seen that significant pitting corrosion was detected on the surfaces of 316L, CoCrMo, and Ti6Al4V alloy. On the contrary, there are not any clear hints of pit formation on TiZrTaHfNb and Ti_1.5_ZrTa_0.5_Hf_0.5_Nb_0.5_ HEA surfaces. Annealing can effectively improve the mechanical properties of the alloy. Researchers have investigated the effect of annealing on the microstructure and properties of titanium based HEA. Nagase et al. (2020) have studied the microstructure of equiatomic and non-equiatomic TiNbTaZrMo HEA, the dendrite coarsening and element segregation can be observed after annealing operation.

**TABLE 2:** Structural features and properties of HEA coatings prepared by arc-melting and powder metallurgy.

Alloy	Procedures	Young’s modulus (Gpa)	Yield strength (Mpa)	Elongation (%)	Hardness (Gpa)	References
Ti-Zr-Nb-Ta	Arc-melting	89	970	23	∼	Yuan et al., 2019
Ti-Zr-Hf-Ta	Arc-melting	86	1367	4.07	∼	Yuan et al., 2019
Ti-Zr-Hf-Nb	Arc-melting	83	728	18.7		Yuan et al., 2019
TiZrHfNbTa	Arc-melting	80	800∼985	∼	3.80	Senkov et al., 2011; Dirras et al., 2016
TiZrNbTaMo	Arc-melting	153	1390	∼	4.90	Wang and Xu, 2017
TiZrHfCrMo	Arc-melting	∼	1250	∼	5.20	Nagase et al., 2020
TiNbTaZrFe	Powder metallurgy	52	2425	∼	0.95	Popescu et al., 2018
TiNbTaZrCr	Arc-melting	∼	∼	∼	6.16	Poletti et al., 2016

As one of the most important methods for preparing HEAs, a class of titanium-based HEAs prepared by arc melting, they believed to have excellent biological properties. However, the cytotoxicity assessment of Ti-based HEAs is still under study, and more *in vivo* and *in vitro* experiments are needed to confirm its biological behavior. Furthermore, mechanical properties are also one of the significant aspects of HEAs in evaluating the functionality of orthopedic materials. The yield strength and Vickers hardness of as-cast titanium-based HEAs are significantly higher than that of 316Lstainless steel, CoCrMo alloy, and Ti6Al4V alloy, which seems promising to increase the longevity of orthopedic implants.

#### Powder Metallurgy

The HEA prepared by powder metallurgy (PM) generally uses elemental powders or pre-alloyed powders as the raw materials (Rao et al., 2014; Wang et al., 2017, 2017a,b). Then, the bulk HEA is prepared through the steps of ball milling/mixing, pressing, sintering, and subsequent processing. Compared with the casting method, PM application can effectively decrease the segregation of alloy components, also it can eliminate the coarse and uneven metal casting structures and significantly improve the optimum raw material consumption (Málek et al., 2019a). However, there are limited studies on the preparation of HEAs by powder metallurgy, at present.

In addition to traditional mechanical processing methods, there are more and more researches and applications of powder metallurgy technology in biomedical metals. According to the recently reported studies about titanium-based HEA alloys, Ti-Nb-Zr, Ti-Mo-Nb, Ti-Nb-Ag, Ti-Nb-Ta-Zr, Ti-Nb-Ta-Mn, Ti-Nb-Ta-V, Ti-Al-Ni-Co-Fe, and Ti-Nb-Hf-Zr-Ta systems (Sakaguchi et al., 2005; Gabriel et al., 2012; Wen et al., 2014; Hussein et al., 2015; Nazari et al., 2015; Aguilar et al., 2016; Anand Sekhar et al., 2019; Guo et al., 2019; Málek et al., 2019a,b) were prepared by PM. Wen et al. (2014) conducted a comparative analysis of Ti-Nb-Ag alloy after vacuum sintering and spark plasma sintering. The sample sintered in the vacuum furnace has some pores, while the samples sintered by SPS showed a dense structure on the surface. Additionally, in order to study the relationship between structure and mechanical properties with sintering time in HEAs prepared by PM, Málek et al. (2019b) found that HfNbTaTiZr alloy had better resistance to grain coarsening after sintering compared with the alloy obtained by arc melting, which had a small amount of porosity after sintering, and the porosity is eliminated in the subsequent heat treatment process. Guo et al. (2019) have studied NbTaTiV alloy prepared by PM, which showed excellent properties, higher hardness of 510 HV, yield strength of 1.37 GPa, and compressive fracture strength of 2.19 GPa at room temperature.

Among these alloys, the Ti-Nb-Ta-Zr system alloys found many applications in the biomedical field (Wang et al., 2009, 2015, 2017; Wei et al., 2011; Liu et al., 2015; Ran et al., 2018; Gu et al., 2019; Hafeez et al., 2019, 2020). Sakaguchi et al. (2005) studied the deformation mechanism of Ti-Nb-Ta-Zr alloys with different Nb contents. At the same time, in order to further explore Ti-based HEA with biomedical prospects, the biological properties of other elements added to Ti-Nb-Ta-Zr HEA were studied by Stráský et al. (2017). Besides, Popescu et al. (2018) prepared a novel Ti_40_Nb_20_Zr_20_Ta_10_Fe_10_ HEA that was showed the outstanding corrosion resistance compared with Ti6Al4V. Cao et al. (2020) prepared TiNbTa_0.5_ZrA_*l0.5*_ HEA by PM and found that after hot-working, the TiNbTa_0.5_ZrA_*l0.5*_ HEA transformed from the initial BCC phase to BCC and HCP phase with 1740 MPa high-pressure yield strength (Chen et al., 2019).

From the above, it can be seen that powder metallurgy also can be an excellent alternative process to produce Ti-based alloys. This method can manufacture porous titanium parts at a lower processing temperature and allows more precise control of process variables and pore size, as well as physical and chemical properties. In the currently existing research, it is found that the Ti-based HEA prepared by powder metallurgy has excellent corrosion resistance in simulated body fluids (SBFs) and may become a potential biomedical substitute. In particular, TiNbZrTaFe HEA has ultra-low Young’s modulus and better corrosion resistance than traditional mental implant materials. Especially with the development of laser 3D printing technology, personalized metal implants will gradually be promoted in clinics application. However, there are not many studies on the preparation of Ti-based HEAs by powder metallurgy, and follow-up on this method will continue to focus on its biomedical research.

### Preparation of Thin Film HEAs

According to the reported methods for preparing HEA coatings, the surface modification techniques include electrochemical deposition (Soare et al., 2015), physical vapor deposition (PVD) (Khan et al., 2020; Xia et al., 2020), laser cladding (Zhang et al., 2017; Guo et al., 2018; Wang L. et al., 2019), plasma cladding (Anupam et al., 2019; Peng et al., 2019; Zhu et al., 2020), and thermal spraying (Wang et al., 2011; Vallimanalan et al., 2020), etc. Particularly, the magnetron sputtering and laser cladding in the biomedical field are two relatively promising technologies for the preparation of HEA coatings. The properties of titanium-based HEA coatings prepared by two different methods are listed in Table 3.

**TABLE 3:** Structural features and properties of HEA coatings prepared by laser cladding and magnetron sputtering.

Alloy	Substrates	Procedures	Phases	Young’s modulus (GPa)	Hardness (GPa)	References
(TiVCrZrHf)N	Si	Magnetron sputtering	FCC	∼267.3	23.8	Liang et al., 2011
(TiZrNbHfTa)C	M2 steel	Magnetron sputtering	FCC	∼	∼28	Braic et al., 2012
(TiZrNbHfTa)N	C45 steel	Magnetron sputtering	FCC	∼	∼33	Braic et al., 2012
TiTaHfNbZr	Ti-6Al-4V	Magnetron sputtering	Amorphous	∼	12.51	Tüten et al., 2019
VAlTiCrCu	Q235 steel	Magnetron sputtering	BCC		12	Chen et al., 2019
(TiHfZrVNb)N	Steel	Cathode vacuum-arc	FCC	∼384	44.3	Pogrebnjak et al., 2014
TiZrNbWMo	45 steel	Laser Cladding	BCC+β-Ti_x_W_1__–__x_	∼	12.74	Zhang et al., 2017
TiAlNiSiV	Ti-6Al-4V	Laser Cladding	BCC	∼∼	11.27∼13.2	Zhang L.-C. et al., 2020
Ni-Cr-Co-Ti-V	Ti-6Al-4V	Laser Cladding	BCC	∼	6.86	Cai et al., 2018

#### Magnetron Sputtering

**FIGURE 5:**
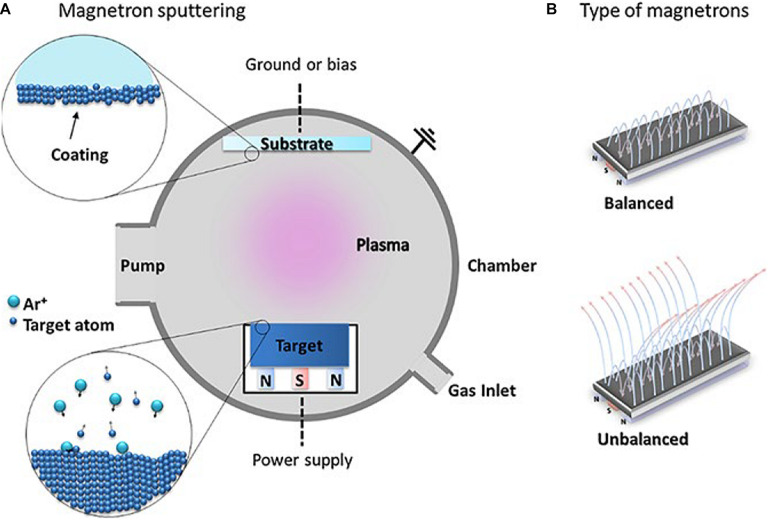
**(A)** The schematic of magnetron sputtering, **(B)** balanced and unbalanced types of magnetron configurations used in MS (Calderon Velasco et al., 2016). Reproduced from Calderon Velasco et al. (2016) with permission.

As a significant surface modification technology, magnetron sputtering has been widely used in various fields. Magnetron sputtering, divided into direct current (DC) magnetron sputtering and radio frequency (RF) magnetron sputtering by the power supply type that is one of the PVD methods (Yan et al., 2018; Deng et al., 2020). This method utilizes the plasma phenomenon to bombard the target material in order to separate metal atoms from the surface of the target and deposit a thin film on the substrate surface. The argon (Ar) and nitrogen (N_2_) gases are often used as the working gases for the preparation of HEA films. A schematic diagram of this process is shown in Figure 5 (Calderon Velasco et al., 2016). The working gas types and its parameters have an extensive impact on the structure and performance of the prepared HEA films.

Cui et al. (2020) studied the effect of different nitrogen contents on the microstructure and mechanical properties of the deposited HEA films, prepared the (AlCrTiZrHf)N film by RF magnetron sputtering. They found that the hardness and friction coefficient of the HEA film changed as the nitrogen flow increased and the film transformed from the initial amorphous state to the columnar structure with the FCC structure, this exhibits the low friction coefficient and excellent wear resistance. Liang et al. (2011) found that in the nitrogen flow rate of 4 SCCM, the hardness and elastic modulus of (TiVCrZrHf)N coatings reach respectively to their maximum values of about 23.8 ± 0.8 and 267.3 ± 4.0 GPa. Furthermore, the corrosion resistance of the NbTiAlSiZrNx HEA coating was prepared by Xing et al. (2019) under the various nitrogen flow rates is significantly different from that of 304 stainless steel. Lukáè et al. (2020) prepared the HfNbTaTiZr HEA film that presents a nano-cell structure with fine surface microstructure and uneven distribution of defects by DC magnetron sputtering. Chen et al. (2019) have studied the mechanical properties and tribo-corrosion behavior of VAlTiCrCu HEA thin film deposited on the surface of 304 stainless steel at different deposition temperatures by magnetron sputtering. The film showed excellent corrosion resistance in the H_2_SO_4_ solution, and the hardness of the film at the deposition temperature of 300°C was 10.93 ± 1.07 GPa, and the elastic modulus was 230.04 ± 56.03 GPa.

Recently, Scholars have discovered that HEA or medium-entropy alloy films are expected to become the potential materials to be used as surface modification coatings on implants (Nguyen et al., 2018; Chen et al., 2020; Wang S. et al., 2020). Chen et al. (2020) prepared TiTaNb medium entropy alloy films by magnetron sputtering, which has excellent bio-corrosion, higher wear resistance, and higher hardness. In addition to spraying or depositing HEA coating on the surface of the implant material, depositing a hydroxyapatite layer on the surface of the implant material can also improve the biocompatibility of metal material by using the magnetron sputtering method. The deposition parameters can be controlled to obtain a thin, flawless, and uniform layer, with tight adhesion to the substrate, low roughness, corrosion and abrasion resistance, which are essential characteristics in medical applications. Despite the higher Young’s modulus of the HEA film compared to human bones, its application as a surface coating does not affect Young’s modulus of the implant material.

#### Laser Cladding

**FIGURE 6:**
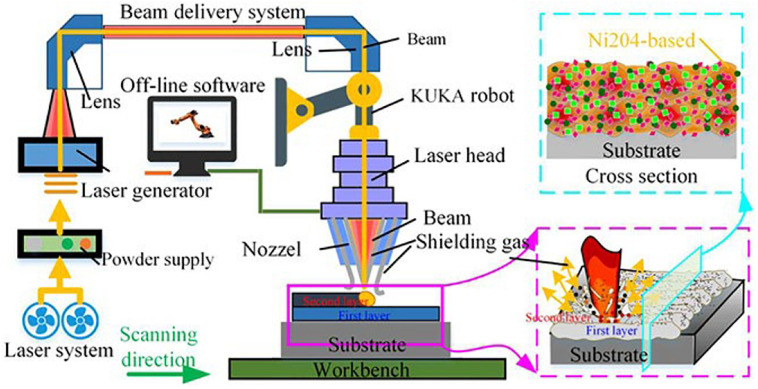
Schematic diagram of the laser cladding (Zhao et al., 2020). Reproduced from Zhao et al. (2020) with permission.

Laser cladding, as a well-known surface modification process, mainly improves the hardness, wear resistance, and corrosion resistance of the surface of the substrate by cladding the alloy powder on the substrate. At present, the common substrate materials which were selected include Q235, Al, titanium, magnesium alloys, and tool steel (Zhang et al., 2017, 2019; Zhang J. et al., 2020; Tian et al., 2019; Wang L. et al., 2019). The principle diagram of laser cladding is shown in Figure 6. Moreover, the fundamental process parameters of laser cladding from several aspects are laser power size, powder feeding method, scanning speed, spot diameter, and overlap ratio (Shu et al., 2019).

In order to study the effect of the Ti element on the wear resistance of the coating prepared by laser cladding, Wang X. et al. (2020) studied CoCrFeNiTix HEA coatings with different Ti content and found that the hardness and corrosion resistance of the coatings increased with the addition of Ti content. Furthermore, the main corrosion mechanism of CoCrFeNiTix high entropy coatings in 3.5wt% NaCl solution is pitting corrosion. Cai et al. (2018) conducted an in-depth analysis of the phase composition and wear resistance of the Ni-Cr-Co-Ti-V HEA coating after the laser surface modification. The coating after the cladding and remelting process contains the Ti-rich phase and BCC phase with the excellent wear resistance. Zhang L.-C. et al. (2020) prepared Ti-based high entropy coatings (TiAlNiSiV) deposited on Ti-6Al-4V alloy, which showed the BCC structure and 1151∼1357 HV hardness in the coating. In addition, the authors analyzed the coating strengthening mechanism and found that solid solution strengthening and the dispersion strengthening are the main reasons for the hardness increase in the coating. Zhang et al. (2017) characterized the microstructure of the TiZrNbWMo HEA coating prepared by laser cladding. The microstructure mainly consists of dendrites and interdendritic structures. Also, through TEM characterization, nano-precipitates were found in this coating that can be explained by the high-entropy and slow diffusion effect of the HEA. Therefore, the combination of laser cladding technology on HEA is a new attempt in surface modification technology to improve the excellent performance of HEAs.

A great deal of research has focused on exploring the effects of surface morphology and composition on implants. The biomimetic design of the coating roughness and porosity without affecting the chemical structure of the substrate can accurately control the mechanical properties and biological reactions of the coating. Laser cladding is a flexible and effective method to obtain the desired properties by mixing different powder materials to form a special biological coating on the surface of the part. However, due to the inconsistency between the thermal expansion coefficient of the cladding layer and the matrix, surface quality problems such as cracks and pores in the cladding layer may be difficult to be accurately controlled.

In recent years, several concerns have been raised on the surface modification of titanium alloy (Liu W. et al., 2019; Wang Q. et al., 2020; Zhang L.-C. et al., 2020), which illustrates that the surface modification technologies play a significant role in improving the surface properties of the implant. In addition, surface modification methods have certain limitations because the antibacterial coatings deposited on the surface of the implant does not have long-term antibacterial performance due to the poor wear resistance and weak bonding forces between the substrates. The titanium-based HEA coatings deposited by laser cladding and magnetron sputtering exhibit good corrosion resistance and wear resistance, hence HEAs can be used as a potential coating on the surface of long-term implants. However, the application of titanium-based HEA coatings in medical implants is still in its infancy, and more *in-vivo* experiments are needed in the later stages to ensure its potential use in cardiovascular or oral implants.

## Biomedical Applications

The biomedical materials can be divided into several groups, including metallic, polymer, ceramic, and bio-composite materials (Chen and Thouas, 2015). Among them, ceramic materials are favored in the clinical use of artificial joints, dental restorations, and cardiovascular system restoration operations due to their good wear properties, chemical stability, high hardness, and good biocompatibility. Biopolymer materials are widely used for drug release carriers, non-permanent implantable devices, tissue induced regeneration, and tissue engineering. In recent years, bioceramics have been widely used in orthopedic clinics due to their excellent biocompatibility, corrosion resistance, and rigidity. Patients can benefit from ceramic components to replace damaged bone tissue and fill bone defects with bioceramic particles. However, in the context of bone tissue engineering, ceramic scaffolds are easy to become brittle, and like metal scaffolds, its degradation rate is also difficult to accurately control. Therefore, the development of ceramic/polymer composite scaffolds with excellent mechanical properties has caused more and more researchers. Pay more attention. On the other hand, the release of ions in ceramic materials can inhibit the inflammatory response of macrophages, which is believed to strongly affect the biological activity of cells and tissue regeneration. The hydrophobicity of the polymer makes it lack of cell adsorption capacity, which limits its application in medical implants without secondary modification (Lozano et al., 2010; Chen and Liu, 2016; Huang et al., 2018). Therefore, a secondary chemical treatment is introduced to treat the surface of the polymer-based scaffold and use organic solvents to increase micropores. Usually, surface modification or the addition of biologically active substances helps cell adhesion and proliferation. Therefore, the development of new biomaterials is very urgent, and the emergence of titanium-based HEAs is expected to meet actual clinical needs. Ilt is very necessary to carry out corresponding *in vivo* and *in vitro* experiments.

### *In-vivo* Evaluation

Before the implant material is officially put into clinical use, it is necessary to evaluate its biological safety both *in vivo* and *in vitro*. The most effective method for biocompatibility assessment is to conduct *in vivo* test but unfortunately, the direct biocompatibility test on the human body is risky, so animal implantation tests are usually used to evaluate the biological safety of materials. In particular, the possible applicability in human tissues should be interpreted with caution because the results of animal models may not necessarily predict the results of human use.

**FIGURE 7:**
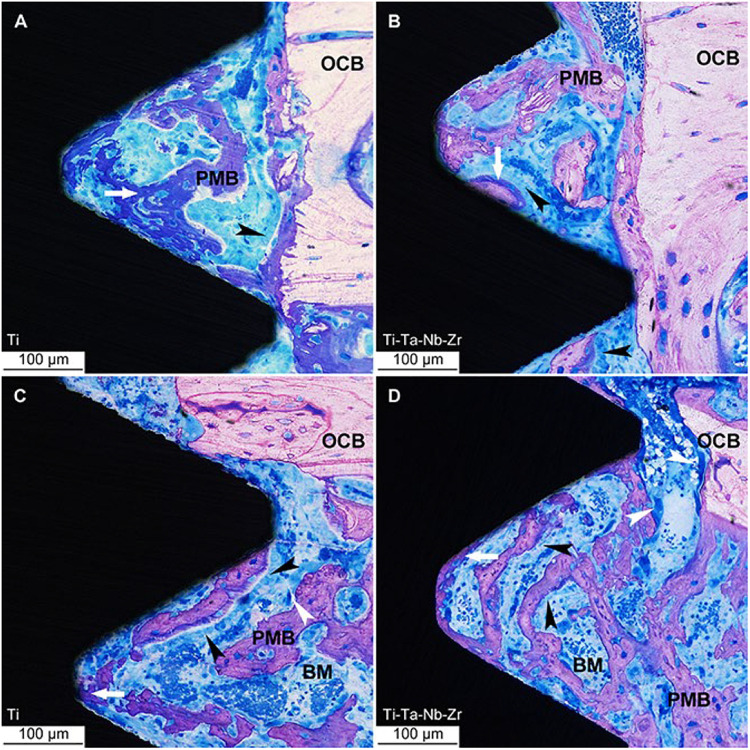
Light micrographs of undecalcified ground sections of bone interface to Ti implants. **(A–C)** and Ti–Ta–Nb–Zr implants **(B–D)** after 7 days’ healing. OCB, original cortical bone; PMB, partially mineralized bone; BM, bone marrow; white arrow = osteoid, white arrowhead = osteoclast and the black arrowheads = osteoblast seams (Stenlund et al., 2015). Reproduced from Stenlund et al. (2015) with permission.

Guo et al. (2013) evaluated the biocompatibility of Ti_35_Nb_2_Ta_3_Zr alloy both *in vivo* and *in vitro* conditions and found that the degree of new bone formation around Ti_35_Nb_2_Ta_3_Zr implants is equivalent to that around Ti6Al4V implants, which showed excellent bone tissue compatibility *in vivo*. Stenlund et al. (2015) evaluated the osseointegration ability of Ti-Nb-Ta-Zr alloy that exhibits the same biological properties with implanted pure titanium in the rat tibial model. The light micrographs of undecalcified ground sections of the bone interface of Ti implants and Ti–Ta–Nb–Zr implants are shown in Figure 7. It could be observed that the osteoblasts arranged as a woven bone in the periphery of implanted material and indicated bone formation.

The medical research on Ti-based HEAs has remained in the material selection stage. As far as the authors of this article know, there is not any comprehensive *in vivo* evaluation study about Ti-based HEAs as implants. *In vivo* tests generally evaluate the following aspects: biomechanics, histology, histomorphometry, and ultrastructure, as well as gene expression. It is necessary to conduct in-depth research on the *in vivo* evaluation of Ti-based HEAs in the future.

### *In-vitro* Evaluation

#### Antibacterial Test

According to reports, titanium alloys and other metallic materials as implants are susceptible to bacterial infections after surgery in the human body. At present, in addition to the use of antibiotics, implants with antibacterial properties are used in order to reduce the rate of bacterial infections during implant repair surgery. Once a bacterial infection occurs, the implant may be loosened from its place, and it may fail. The patient usually needs to take antibiotics for a long time or even undergo multiple operations to heal, which will increase the mental and financial burden for both patients and the medical system. Therefore, biomedical material researchers have been committed to developing some new materials with antibacterial function. The medical field also urgently needs to develop new biomedical materials that can play a long-term anti-infection function, thereby reducing the possibility of infection and reducing the abuse of antibiotics. It is of far-reaching significance to alleviate the suffering of patients and improve people’s quality of life. In the previous research literature (Wu et al., 2006), some researchers tested the antibacterial properties of HEA by inoculating *Staphylococcus aureus*, *Escherichia coli*, *Klebsiella pneumoniae*, and *Pseudomonas aeruginosa* bacteria on the surface of HEA coating. After 24 h, it was found that the HEA coating had a significant inhibitory effect on the bacterial colony formation, and the antibacterial rate reached 99.99%, which indicated that the HEA coatings had positive antibacterial properties.

**FIGURE 8:**
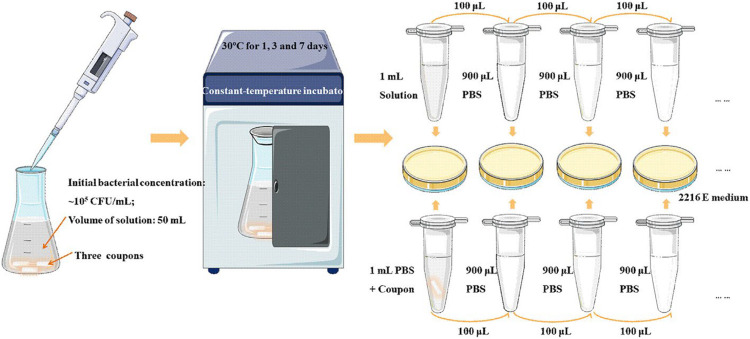
Schematic diagram displaying the antibacterial testing experimental process (Zhou et al., 2020). Reproduced from Zhou et al. (2020) with permission.

**FIGURE 9:**
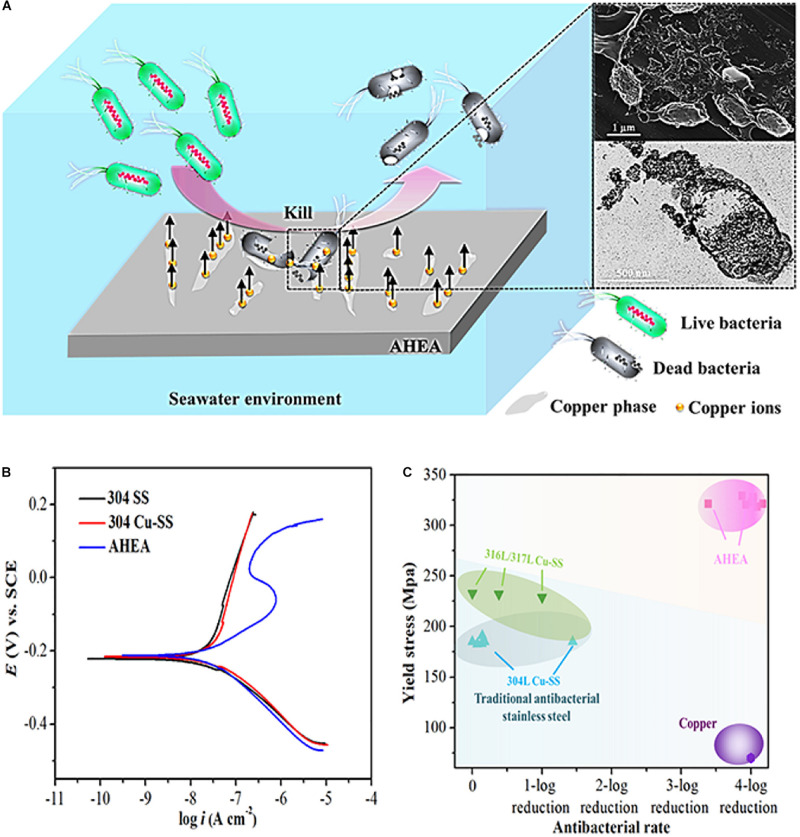
**(A)** Schematic illustration for the antimicrobial mechanism of AHEA, **(B)** polarization curves of 304 SS, 304 Cu-SS, and AHEA in the simulated seawater and **(C)** yield stresses and antibacterial rates (Zhou et al., 2020). Reproduced from Zhou et al. (2020) with permission.

Generally, a small number of antibacterial elements such as Cu and Ag will be added to the antibacterial metal material to enhance its mechanical properties and the antibacterial activity (Liu et al., 2014; Han et al., 2020; Wang Y. et al., 2020). In recent years, some scholars have studied the antibacterial properties of copper-containing Ti-based HEAs and copper-containing HEAs. Ke et al. (2019) prepared a medical Ti-13Nb-13Zr-10Cu alloy that its elastic modulus was significantly lower than CP-Ti, and showed significant antibacterial activity after 24 h of *S. aureus* culture. In addition, Zhou et al. (2020) designed a novel Cu-bearing Al_0.4_CoCrCuFeNi high-entropy alloy (AHEA) that was used to prevent the growth of bio-corrosive marine bacteria. At the same time, the antibacterial properties of different samples (HEA and 304 stainless steel, and copper-containing 304 stainless steel and pure copper) were compared by three marine bacteria. The antibacterial test process is shown in Figure 8. After 1, 3, and 7 days of cultivation in the medium, the bacterial colonies on the HEA have significantly reduced, and the antibacterial effect was similar to pure copper (Figure 9).

#### Electrochemical Test

In recent years, due to the widespread use of metallic implant materials in the medical field, researchers have tried to observe and investigate patients after repair surgery. They found that the implant material can be corroded due to complex body fluids interaction. Also, stress wear between bones can happen. As a consequence, the material will plastically deform, and its properties are affected finally, the implant will fail, and the patients may undergo multiple repair operations in severe cases. At the same time, the implant materials wear with the surrounding tissues of the human body leads to the formation of metallic debris formation that may cause tissue infection and allergic effects. Furthermore, corrosion and stress wear of implants can cause the precipitation of some toxic metal ions, which in turn can trigger toxic and allergic reactions. In order to reduce the harmful effects of implants in the human body, the production of novel implant materials with excellent comprehensive performance is necessary. Therefore, in the current research of implant materials, the corrosion resistance and friction and wear properties of implants are a focus of researchers (Ghiban et al., 2018).

Due to a certain effect of the pH value of the simulated physiological environment on the corrosion behavior of metal implants, many researchers have explored the corrosion behavior of titanium-based HEAs as potential implant materials in simulated physiological environments. Song and Xu (2020) investigated the (TiZrNbTa)_90_Mo_10_ HEA electrochemical properties in Ringer’s solution and used XPS to characterize the passive film formation on the surface of (TiZrNbTa)_90_Mo_10_ HEA. In addition, (TiZrNbTa)_90_Mo_10_ HEA exhibits robust corrosion resistance far better than CoCrMo alloys and stainless steel. Braic et al. (2012) prepared (TiZrNbHfTa)N and (TiZrNbHfTa)C coatings by DC magnetron sputtering on Ti6Al4V alloy. They examined the processed samples by the corrosion and tribological tests in SBFs, as well as cell viability tests. These films exhibited good protective properties and did not induce any cytotoxic response by osteoblasts (24 and 72 h), with good morphology of the attached cells. Aksoy et al. (2019) have studied the deposition of TiTaHfNbZr HEA films on NiTi shape memory alloy by RF magnetron sputtering, then they immersed it into artificial saliva (AS) and gastric fluid (GF) solutions. This study demonstrates that TiTaHfNbZr HEA coatings have a significant inhibitory effect toward the release of Ni ions. Therefore, TiTaHfNbZr HEA thin films can serve as a potential biomedical coating on NiTi implants to prevent the release of Ni ions. Tüten et al. (2019) prepared TiTaHfNbZr HEA coatings on Ti-6Al-4V substrates by RF magnetron sputtering, which are considered an effective coating for long-term orthopedic implants with a protective effect on surface wear and cracking.

#### Cell Adhesion and Cytotoxicity Assay

Cell adhesion is the basic condition for maintaining the stability of tissue structure, and it is also the regulator factor of cell movement and function that has a significant influence on cell proliferation and differentiation (Yang et al., 2011). Cytotoxicity assay is usually used to evaluate the effect of alloy on cell growth activity that represents one of the most key indicators in the *in vitro* evaluation (Rincic Mlinaric et al., 2019). Cytotoxicity is the process of chemical substances, drugs, or physiological action of cells acting on the basic structure. These processes include cell membrane or cytoskeleton structure, cell metabolism process, synthesis, degradation, or release of cell components or products, ion regulation, and cell division, etc. finally leading to cell survival, proliferation, or functional disorders that result in adverse reactions.

In recent years, researchers have found in the follow-up study of patients after implant repair surgery that long-term implantation of metal materials in the human body will cause a series of biological problems. For example, the metal elements such as Co and Ni in cobalt-based alloys have serious sensitization problems, as well as the long-term implantation of Al and V elements in the commonly used Ti-6Al-4V implants will exert influence onhuman organs and functions. Therefore, the detection of cytotoxicity and the activity of metal implant materials is of great significance before implantation and repair surgery in humans. Cytotoxicity and cell activity assays are mainly based on the function of individual cells and they can change in cell membrane permeability. According to scholars’ previous research, cytotoxicity and activity detection methods are divided into the following four types: dye exclusion assays, colorimetric assays, fluorometric assays, luminometric assays (Aslantürk, 2018). Among them, MTT assay, XTT assay, LDP assay of colorimetric assays are among the most commonly used techniques in cell detection.

**FIGURE 10:**
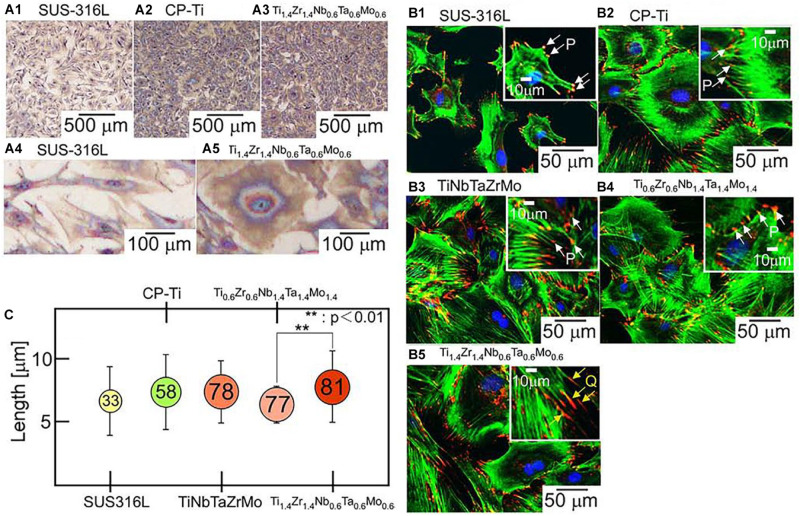
Biocompatibility of the ingots of bio-HEAs. **(A)** Giemsa staining images of osteoblasts on the fabricated specimens of SUS316L (stainless steel), CP-Ti (commercial pure titanium), and Ti_1.4_Zr_1.4_Nb_0.6_Ta_0.6_Mo_0.6_, **(B)** fluorescent images of osteoblast adhesion on the fabricated specimens of SUS316, CP-Ti, equiatomic TiNbTaZrMo, and non-equiatomic Ti_2–x_Zr_2–x_Nb_x_Ta_x_Mo_x_ (*x* = 0.6, 1.4) bio-HEAs, and **(C)** quantitative analysis of size regulation of fibrillar adhesions (longer than 5 μm) in osteoblasts cultured on the fabricated specimens (Hori et al., 2019). Reproduced from Hori et al. (2019) with permission.

In order to evaluate the adhesion effect of osteoblasts on the surface of HEA immunocytochemical methods were used to observe the cell adhesion behavior. Hori et al. (2019) designed a series of novel non-equiatomic Ti-Nb-Ta-Zr-Mo HEAs. They demonstrated that this HEA system promoted the maturation of local adhesion spots in osteoblasts. As shown in Figure 10, it can be seen that the number of osteoblasts adhesion on the two HEAs, including Ti_1.4_Zr_1.4_Nb_0.6_Ta_0.6_, and Ti_0.6_Zr_0.6_Nb_1.4_Ta_1.4_Mo_1_._4_ is larger than SUS316L. Moreover, Ti_1.4_Zr_1.4_Nb_0.6_Ta_0.6_Mo_0.6_ shows superior biocompatibility because of its fibric adhesion structure is significantly longer than Ti_0.6_Zr_0.6_Nb_1.4_Ta_1.4_Mo_1_._4_. Nagase et al. (2020) found that Ti–Zr-Hf-Cr-Mo and Ti–Zr-Hf-Co-Cr-Mo HEAs showed excellent biocompatibility compared with CP-Ti. Edalati et al. (2020) have studied TiAlFeCoNi HEA exhibited ultra-high hardness and favorable cellular activity by a combination of MTT assay and microhardness measurements. Todai et al. designed a new TiNbTaZrMo HAE that shows good biocompatibility compares to Cp-Ti. The osteoblasts on the as-cast and annealed TiNbTaZrMo HEA surface show a wide range of morphology, which is similar to the osteoblasts on the Cp-Ti surface. On the other hand, the distribution of osteoblasts on 316L stainless steel shows a smaller number of various morphologies. Osteoblasts on TiNbTaZrMo HEAs and Cp-Ti are very advantageous in the bone matrix formation.

It can be understood from the above-mentioned explains that Ti-based HEAs have a great potential to become a biomedical material due to their excellent antibacterial ability, wear resistance, and corrosion resistance, as well as lower cytotoxicity. In particular, Ti-Nb-Ta-Zr HEAs and TiTaHf-based HEAs have considerable biocompatibility compared with Cp-Ti and attracts lots of attention from research communities. Generally, implantable devices made of biomaterials aim to improve the quality of life and extend the life of patients. After long-term use of plastic surgical prostheses made of biologically inert materials, the current focus is on materials that promote the proliferation and differentiation of osteoblasts and activate tissue repair mechanisms (called biologically active materials). In addition, regarding the biocompatibility of implantable alloys, it is necessary to ensure improved corrosion resistance in corrosive physiological environments (Yang et al., 2020). Therefore, more attention should be focused on the Ti-Nb-Ta-Zr system HEAs in the future. Furthermore, the HEA design is also a critical factor in determining its performance, and researchers should focus on the establishment of the proper design principles and criteria to develop the novel bio-HEAs.

## Conclusion

In this review, an insight into the development of Ti-based HEAs is provided, and it summarizes the current methods in HEAs fabrication, HEA blocks or coatings, and analysis its properties and biological applications. Metallic implant materials usually are made of traditional titanium alloy, 316L stainless steel, and CoCrMo alloys, and they have less harmful effects on the human body after the surgery. In order to develop implant materials with excellent functional properties, many scholars began to turn their attention to HEAs, which have a wide range of applications resulting from its exceptional physical, chemical, magnetization, and mechanical properties. The emergence of HEAs has brought great room for development in the field of medical implant materials. This HEA design concept overturns the principles of traditional alloy design, and it emphasizes multiple principal elements as the basis. Also, a small number of modified elements were utilized to mutually control the structure and mechanical properties of the alloy.

## Prospects

In recent years, due to the continuous improvement of medical science and technology, the performance requirements of implant materials for revision and implantation operations have become more and more significant. Ti-based HEAs have recently emerged as alternative implant materials to address some of the unresolved issues in terms of performance and biocompatibility. Compared with traditional alloys, the complexity of various chemical elements in HEAs makes them functional. The combination of chemical elements can induce excellent mechanical properties also it ensures functionality and biocompatibility. Moreover, it is suitable for use as a new type of biocompatible metal material. Although titanium-based HEAs are among the new potential metallic implant material, its cytotoxicity and biological evaluation and research on its implantation in animals are in the initial stage and Ti-based HEAs still have not been used clinically. Therefore, the future development of titanium-based HEAs still needs a lot of experimental research and further in-depth exploration.

## Author Contributions

NM wrote the main part of the manuscript. SL and WL greatly contributed to the fabrication methods parts. NM and BZ made a contribution to data curation. LX, DW, LW, and YW made significant contributions particularly in revising the manuscript. NM, LL, and WL prepared and formulated the references. All the authors contributed to the manuscript and approved the submitted version.

## Conflict of Interest

LL and BZ were employed by the company Chengsteel Group Co., Ltd., HBIS Group Co., Ltd. The remaining authors declare that the research was conducted in the absence of any commercial or financial relationships that could be construed as a potential conflict of interest.
